# Sex disparities in current cigarette smoking prevalence among individuals aged 15 years and older in 39 African countries: a descriptive study using the World Health Organization Health Equity Assessment Toolkit

**DOI:** 10.1080/16549716.2026.2728849

**Published:** 2026-09-08

**Authors:** Augustus Osborne, Umaru Sesay, Abdirasak Sharif Ali

**Affiliations:** a Institute for Development, Freetown, Sierra Leone; b Division of Disease Control and Prevention, Africa CDC, Addis Ababa, Ethiopia; c Department of Microbiology and Laboratory Sciences, Faculty of Medicine and Health Sciences, SIMAD University, Mogadishu, Somalia

**Keywords:** Health inequality monitoring, male-female differences, non-age-standardized estimates, public health surveillance, tobacco policy

## Abstract

**Background:**

Cigarette smoking remains a major preventable cause of disease and mortality. Comparable sex-disaggregated estimates are needed to describe smoking patterns across African countries and support tobacco control planning.

**Objective:**

To provide a multi-country descriptive summary of the current prevalence of cigarette smoking disaggregated by sex among people aged ≥ 15 years in 39 African countries.

**Methods:**

This cross-sectional descriptive analysis used non-age-standardized WHO Global Health Observatory modelled estimates for 2022, accessed through Health Equity Assessment Toolkit version 6.0. Current cigarette smoking prevalence (M_Est_cig_curr) was disaggregated by sex and exported for 39 countries. Absolute differences were calculated as female minus male prevalence. Male-to-female prevalence ratios were derived from the exported point estimates for supplementary analysis. The dataset comprised modelled estimates rather than directly observed survey data.

**Results:**

The overall prevalence ranged from 1.7% in Ghana to 18.2% in Seychelles. The prevalence of males exceeded that of females in all 39 countries. The male-minus-female gap ranged from 3.3 pp in Ghana to 36.4 pp in Lesotho; gaps also exceeded 25 pp in Mauritius, Algeria, Madagascar, and Seychelles. The prevalence in females was highest in South Africa (5.1%) and Seychelles (4.5%).

**Conclusion:**

This study shows substantial sex differences in current cigarette smoking across the included African countries, driven mainly by a higher prevalence among men. These findings support strengthened sex-disaggregated surveillance and tobacco control responses tailored to observed country patterns. Explanations involving social norms, marketing, or policy context should be treated as hypotheses because these factors were not measured in this study.

## Paper Context

- **Main findings:** Across 39 African countries, the modelled prevalence of cigarette smoking varied widely and was higher among males than females in every setting.
- **Added knowledge:** A common reference year and standardized source reveal where absolute male-female differences are largest, while showing the instability of relative ratios when female prevalence is very low.
- **Global health impact for policy and action:** Sex-disaggregated surveillance can help countries prioritize tobacco control responses without treating unmeasured cultural, marketing, or policy factors as established causes.

## Background

Tobacco use remains one of the leading preventable causes of morbidity and mortality worldwide, and cigarette smoking is associated with cardiovascular disease, cancer, respiratory illness, and other adverse health outcomes [1–3]. In low- and middle-income countries, public health agencies and researchers have raised concerns about tobacco industry marketing and the uneven implementation of tobacco control measures [3,4]. Comparable sex-disaggregated data are needed to describe smoking patterns and guide interventions without assuming that the same contextual factors operate in every setting.

Global estimates consistently show a substantially higher smoking prevalence among men than among women [5,6]. Although the prevalence has declined in many settings, projections indicate that cigarette demand and the number of users may remain important public health concerns in Africa because of population growth and changing tobacco markets [4,7]. World Health Organization reports also show substantial variations in tobacco control implementation and smoking prevalence across African countries [4,6]. These patterns justify standardized country-level comparisons but do not identify the causes of between-country differences.

Previous studies have documented associations between tobacco use and age, sex, education, socioeconomic position, and urban or rural residence [8–13]. However, much of the evidence comes from individual countries, subregions, or global analyses, and comparable sex-disaggregated estimates are not available for every African country. Methodological differences and data gaps complicate cross-country interpretations [14].

The African context is heterogeneous, and smoking patterns may reflect differences in urbanization, income, tobacco control implementation, social acceptability, and tobacco industry activity. Prior literature has discussed cultural and religious norms that may discourage smoking among women in some settings, while also documenting changes in gender norms and tobacco marketing [13–16]. These mechanisms are plausible contextual explanations, but country-level prevalence data alone cannot establish whether they caused the observed patterns in this study.

In this manuscript, sex refers to the male and female categories reported in the World Health Organization dataset and is a measured dimension used for quantitative comparisons. Gender refers to socially constructed roles, norms, and expectations. Gender-related mechanisms, including masculinity, stigma, and marketing, are discussed only as possible interpretations from prior literature and were not measured or tested in this analysis.

This study presents a multi-country descriptive summary of the current prevalence of cigarette smoking in 39 African countries using standardized World Health Organization Global Health Observatory estimates accessed through the Health Equity Assessment Toolkit. The objectives were to describe the overall and sex-disaggregated prevalence, quantify absolute and relative male-female differences, identify countries with the largest observed gaps, and document limitations in the available cross-country surveillance data.

## Methods

### Study design and period

This study used a cross-sectional descriptive design to summarize the current prevalence of cigarette smoking for a single reference year. All included observations were World Health Organization modelled estimates for the 2022 reference year. The value 2022 is therefore not the year of a single country survey; the World Health Organization modelling process draws on available national survey observations from different years to produce comparable country-year estimates. Thirty-nine African countries were included because complete male and female estimates were available in the extracted datasets.

### Data source

Data were exported from the World Health Organization Health Equity Assessment Toolkit, built-in database edition, version 6.0, on 17 June 2025. The dataset source identified in the export was the World Health Organization Global Health Observatory. The selected indicator was M_Est_cig_curr, ‘Estimate of current cigarette smoking prevalence (%)’ for people aged 15 years and older. This is a non-age-standardized model estimate, not a direct estimate from a single survey conducted in 2022.

The Health Equity Assessment Toolkit provides access to pre-processed disaggregated data and summary measures and supports their presentation in tables, charts, and maps. For this indicator, the underlying evidence originates from population-based surveys used in the World Health Organization’s tobacco prevalence estimation process. Survey design procedures, such as sampling weights and stratification, belong to the source surveys and World Health Organization pre-processing or modelling; the authors did not independently reweight survey records or adjust for sampling bias.

Of the 54 African countries, 39 had complete male and female estimates in the exported Health Equity Assessment Toolkit dataset and were included. Countries without complete sex-disaggregated estimates were excluded from the study. No missing values were imputed in this study.

### Outcome measure and dimension of inequality

The primary outcome was the estimated percentage of people aged ≥ 15 years who currently smoked cigarettes, including daily and non-daily cigarette smoking, as defined by the World Health Organization indicator. Estimates were reported for females, males, and the setting average, with lower and upper uncertainty bounds supplied in the export.

Sex was the dimension of inequality. The Health Equity Assessment Toolkit difference measure (D) was interpreted using the exported subgroup order and was defined as female prevalence minus male prevalence; therefore, negative values indicated a higher prevalence among males. To complement the absolute measure, the authors calculated a male-to-female prevalence ratio from the exported point estimates. Ratios were not calculated where the female point estimate was 0.0% because division by zero is undefined, and all ratios based on a very low female prevalence were interpreted cautiously.

### Data analysis

The Compare Inequality component of Health Equity Assessment Toolkit version 6.0 was used to select the World Health Organization Global Health Observatory source, indicator M_Est_cig_curr, sex dimension, and 2022 reference year. The toolkit generated the setting average, sex-disaggregated estimates, lower and upper bounds, and absolute difference (D). The authors exported these outputs, checked country completeness, organized the results, and produced descriptive summaries. No regression, hypothesis testing, survey weighting, or statistical modelling was performed by the authors.

### Ethical considerations

This analysis used publicly available, aggregate World Health Organization estimates and did not involve access to individual-level records. Institutional ethics approval and individual consent were therefore not required.

## Results

### Prevalence of current cigarette smoking in Africa

**Figure 1.**
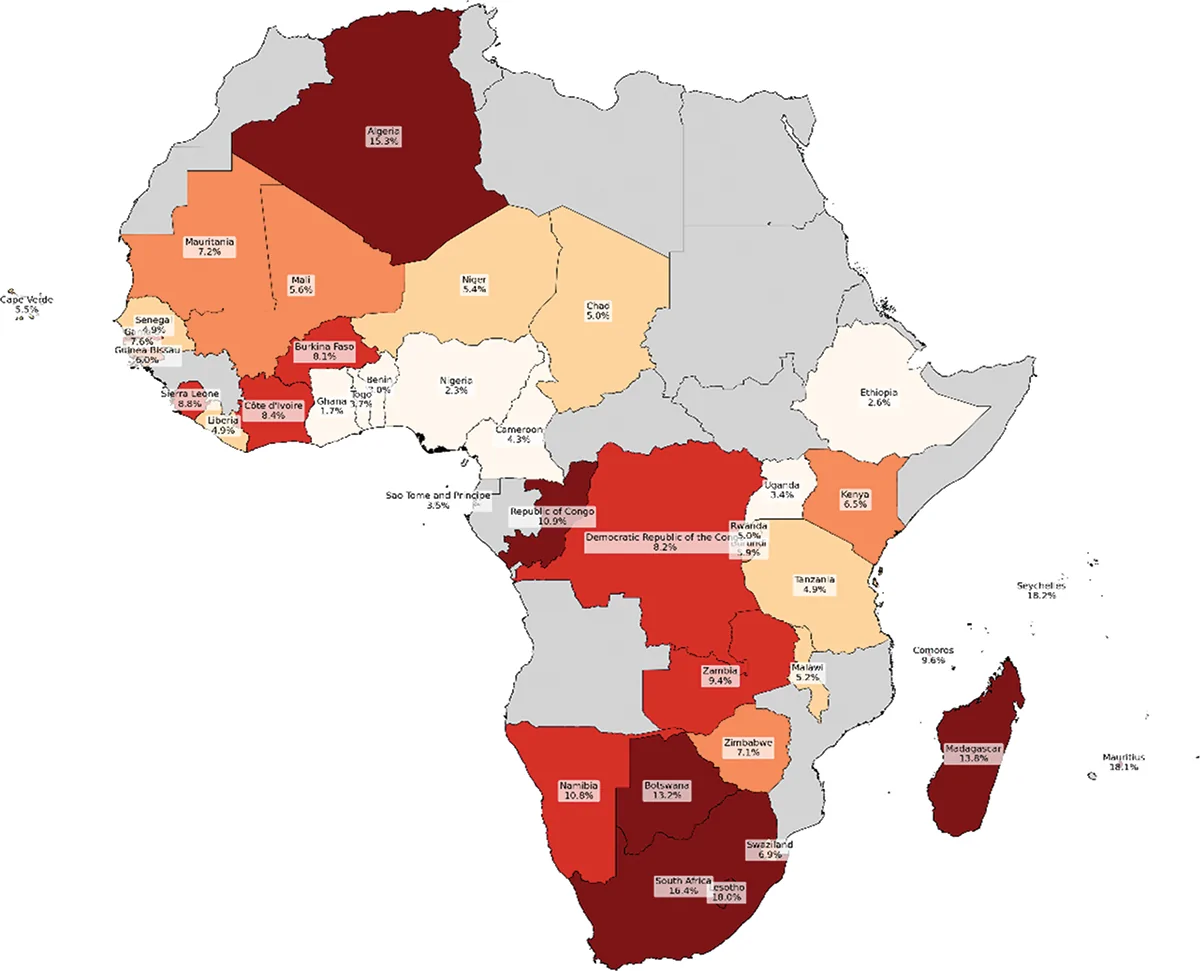
Estimate of current cigarette smoking prevalence in Africa (World Health Organization modelled estimates, reference year 2022).

Across the 39 countries, the setting-average estimate ranged from 1.7% in Ghana to 18.2% in Seychelles (Table 1). Mauritius (18.1%), Lesotho (18.0%), South Africa (16.4%) and Algeria (15.3%) were also among the highest-prevalence settings, whereas Nigeria (2.3%), Ethiopia (2.6%) and Benin (3.0%) were among the lowest, see Table 1 and Figure 1.

**Table 1.** World Health Organization modelled estimate of current cigarette smoking prevalence in 39 African countries, reference year 2022.

Country	Setting-average prevalence (%)
Lesotho	18
Algeria	15.3
Benin	3
Botswana	13.2
Burkina Faso	8.1
Burundi	5.9
Cabo Verde	5.5
Cameroon	4.3
Chad	5
Comoros	9.6
Congo	10.9
Côte d’Ivoire	8.4
Democratic Republic of the Congo	8.2
Eswatini	6.9
Ethiopia	2.6
Gambia	7.6
Ghana	1.7
Guinea-Bissau	6
Kenya	6.5
Liberia	4.9
Madagascar	13.8
Malawi	5.2
Mali	5.6
Mauritania	7.2
Mauritius	18.1
Namibia	10.8
Niger	5.4
Nigeria	2.3
Rwanda	5
Sao Tome and Principe	3.5
Senegal	4.9
Seychelles	18.2
Sierra Leone	8.8
South Africa	16.4
Togo	3.7
Uganda	3.4
United Republic of Tanzania	4.9
Zambia	9.4
Zimbabwe	7.1

Note: Values are non-age-standardized World Health Organization Global Health Observatory modelled estimates for the 2022 reference year. They are not country-specific survey-year observations.

### Sex-disaggregated current cigarette smoking prevalence

Male prevalence exceeded female prevalence in every included country (Table 2). The prevalence of males was highest in Lesotho (36.6%), Mauritius (34.3%), Seychelles (30.3%), Algeria (29.9%), and South Africa (28.7%). The prevalence in females was generally low and highest in South Africa (5.1%), Seychelles (4.5%), Namibia (3.3%), Sierra Leone (2.8%), and Mauritius (2.5%). The lower and upper bounds were wide in several high-prevalence countries, indicating uncertainty in some country estimates, see Table 2.

**Table 2.** Sex-disaggregated World Health Organization modelled estimates of current cigarette smoking prevalence in 39 African countries, reference year 2022.

Reference year	Country	Sex	Estimate (%)	Lower bound	Upper bound
2022	Lesotho	Female	0.2	0.1	0.3
2022	Lesotho	Male	36.6	26.9	46.3
2022	Algeria	Female	0.3	0.2	0.5
2022	Algeria	Male	29.9	21.8	37.9
2022	Benin	Female	0.4	0.3	0.6
2022	Benin	Male	5.6	4.4	6.9
2022	Botswana	Female	2.1	1.4	2.9
2022	Botswana	Male	24.7	17.7	31.8
2022	Burkina Faso	Female	0.5	0.1	0.8
2022	Burkina Faso	Male	16	10.6	21.4
2022	Burundi	Female	0.8	0.5	1.1
2022	Burundi	Male	11.2	6.9	15.6
2022	Cabo Verde	Female	1.1	0.7	1.5
2022	Cabo Verde	Male	10	7.1	12.9
2022	Cameroon	Female	0.2	0.1	0.3
2022	Cameroon	Male	8.4	5.9	10.9
2022	Chad	Female	0.5	0.3	0.6
2022	Chad	Male	9.7	6.9	12.5
2022	Comoros	Female	1.3	0.6	2
2022	Comoros	Male	17.9	10.5	25.3
2022	Congo	Female	0.4	0.2	0.6
2022	Congo	Male	21.6	13.2	29.9
2022	Côte d’Ivoire	Female	0.2	0.1	0.3
2022	Côte d’Ivoire	Male	16.4	11	21.8
2022	Democratic Republic of the Congo	Female	0.3	0.2	0.5
2022	Democratic Republic of the Congo	Male	16.4	9.9	22.8
2022	Eswatini	Female	0.5	0.2	0.8
2022	Eswatini	Male	13.5	8.7	18.3
2022	Ethiopia	Female	0.4	0.2	0.5
2022	Ethiopia	Male	4.8	3.6	6
2022	Gambia	Female	0.2	0.1	0.2
2022	Gambia	Male	15.3	11.4	19.2
2022	Ghana	Female	0.1	0	0.1
2022	Ghana	Male	3.4	2.3	4.5
2022	Guinea-Bissau	Female	0.3	0.2	0.5
2022	Guinea-Bissau	Male	12	7.9	16.1
2022	Kenya	Female	0.3	0.2	0.4
2022	Kenya	Male	12.9	9.5	16.2
2022	Liberia	Female	0.5	0.3	0.7
2022	Liberia	Male	9.5	6	13
2022	Madagascar	Female	0.9	0.7	1.2
2022	Madagascar	Male	26.8	21	32.6
2022	Malawi	Female	0.3	0.1	0.4
2022	Malawi	Male	10.7	8.3	13.1
2022	Mali	Female	0.3	0.1	0.4
2022	Mali	Male	10.8	8.4	13.2
2022	Mauritania	Female	1.5	1	2
2022	Mauritania	Male	13.4	8.3	18.4
2022	Mauritius	Female	2.5	1.6	3.5
2022	Mauritius	Male	34.3	22.4	46.2
2022	Namibia	Female	3.3	2.1	4.6
2022	Namibia	Male	19.2	13.4	25
2022	Niger	Female	0	0	0
2022	Niger	Male	10.6	6.7	14.6
2022	Nigeria	Female	0.2	0.1	0.2
2022	Nigeria	Male	4.5	3.5	5.6
2022	Rwanda	Female	0.7	0.3	1.1
2022	Rwanda	Male	9.6	5.8	13.4
2022	Sao Tome and Principe	Female	0.4	0.2	0.6
2022	Sao Tome and Principe	Male	6.7	4	9.3
2022	Senegal	Female	0.3	0.2	0.4
2022	Senegal	Male	9.9	7.6	12.3
2022	Seychelles	Female	4.5	2.6	6.3
2022	Seychelles	Male	30.3	19.9	40.7
2022	Sierra Leone	Female	2.8	2	3.7
2022	Sierra Leone	Male	14.7	11.4	18.1
2022	South Africa	Female	5.1	3.7	6.6
2022	South Africa	Male	28.7	19.9	37.5
2022	Togo	Female	0.1	0.1	0.2
2022	Togo	Male	7.2	4.9	9.5
2022	Uganda	Female	0.6	0.4	0.7
2022	Uganda	Male	6.5	4.9	8
2022	United Republic of Tanzania	Female	0.4	0.2	0.6
2022	United Republic of Tanzania	Male	9.7	7	12.5
2022	Zambia	Female	0.8	0.5	1
2022	Zambia	Male	18.4	14.7	22.1
2022	Zimbabwe	Female	0.2	0.1	0.3
2022	Zimbabwe	Male	15.4	10.3	20.4

Note: The lower and upper bounds are those provided in the Health Equity Assessment Toolkit export. The reference year is 2022 for every country because these are World Health Organization (WHO)-modelled country-year estimates, not direct observations from surveys conducted in 2022.

### Absolute sex differences in current cigarette smoking prevalence

**Figure 2.**
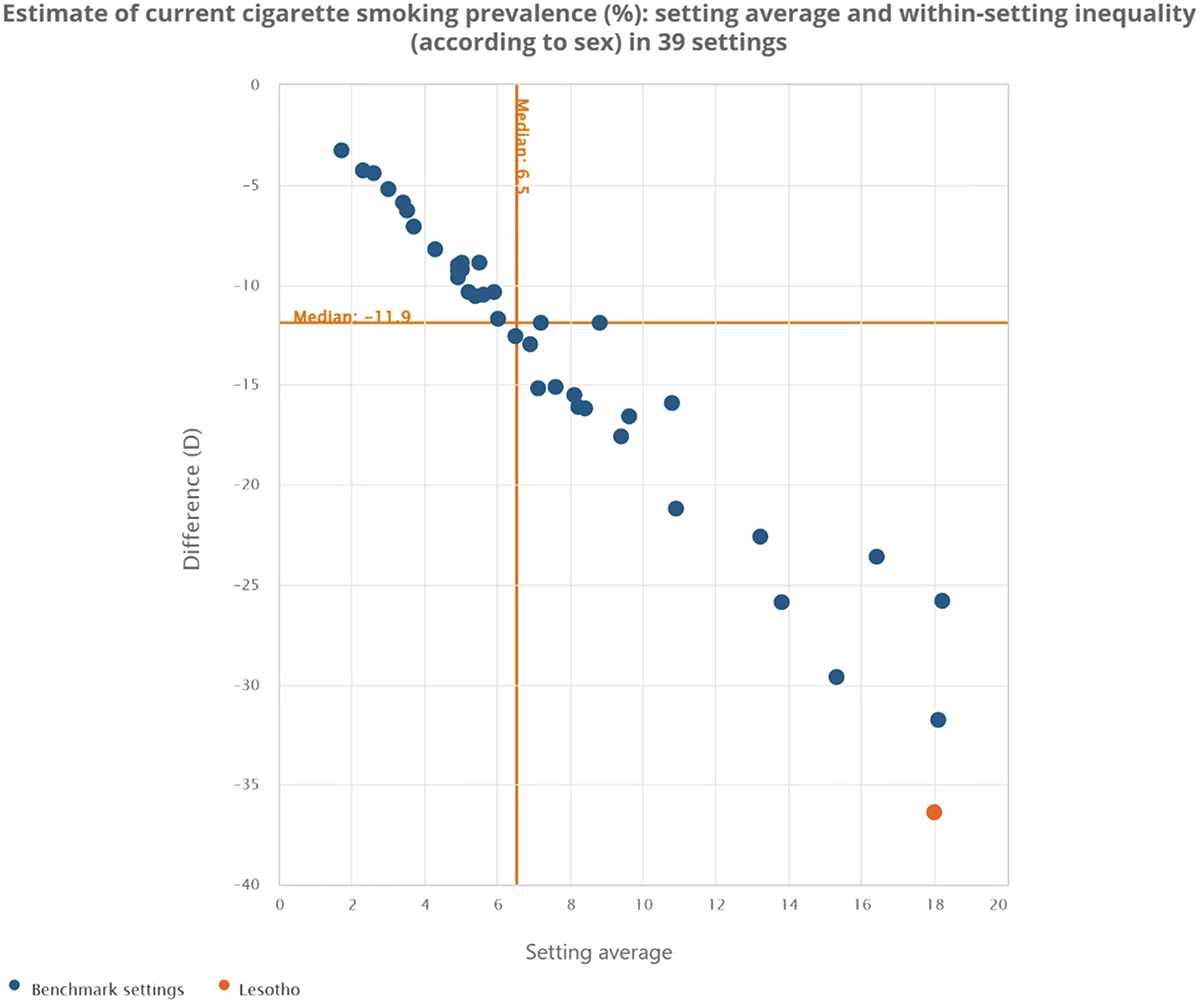
Health Equity Assessment Toolkit: absolute difference in current cigarette smoking prevalence by sex, reference year 2022.

The Health Equity Assessment Toolkit difference (female minus male) was negative in all countries (Table 3 and Figure 2). In absolute magnitude, the largest gaps were observed in Lesotho (36.4% points), Mauritius (31.8), Algeria (29.6), Madagascar (25.9) and Seychelles (25.8). The smallest gaps were in Ghana (3.3), Nigeria (4.3), Ethiopia (4.4), Benin (5.2), and Uganda (5.9). Male-to-female prevalence ratios are reported in Supplementary Table S1; these ratios are highly sensitive to very low female estimates and are therefore secondary to absolute differences, see Table 3 and Figure 2.

**Table 3.** Health Equity Assessment Toolkit absolute difference in current cigarette smoking prevalence by sex, reference year 2022.

Reference year	Country	Measure	Estimate (percentage points)
2022	Lesotho	Difference (D)	−36.4
2022	Algeria	Difference (D)	−29.6
2022	Benin	Difference (D)	−5.2
2022	Botswana	Difference (D)	−22.6
2022	Burkina Faso	Difference (D)	−15.5
2022	Burundi	Difference (D)	−10.4
2022	Cabo Verde	Difference (D)	−8.9
2022	Cameroon	Difference (D)	−8.2
2022	Chad	Difference (D)	−9.2
2022	Comoros	Difference (D)	−16.6
2022	Congo	Difference (D)	−21.2
2022	Côte d’Ivoire	Difference (D)	−16.2
2022	Democratic Republic of the Congo	Difference (D)	−16.1
2022	Eswatini	Difference (D)	−13
2022	Ethiopia	Difference (D)	−4.4
2022	Gambia	Difference (D)	−15.1
2022	Ghana	Difference (D)	−3.3
2022	Guinea-Bissau	Difference (D)	−11.7
2022	Kenya	Difference (D)	−12.6
2022	Liberia	Difference (D)	−9
2022	Madagascar	Difference (D)	−25.9
2022	Malawi	Difference (D)	−10.4
2022	Mali	Difference (D)	−10.5
2022	Mauritania	Difference (D)	−11.9
2022	Mauritius	Difference (D)	−31.8
2022	Namibia	Difference (D)	−15.9
2022	Niger	Difference (D)	−10.6
2022	Nigeria	Difference (D)	−4.3
2022	Rwanda	Difference (D)	−8.9
2022	Sao Tome and Principe	Difference (D)	−6.3
2022	Senegal	Difference (D)	−9.6
2022	Seychelles	Difference (D)	−25.8
2022	Sierra Leone	Difference (D)	−11.9
2022	South Africa	Difference (D)	−23.6
2022	Togo	Difference (D)	−7.1
2022	Uganda	Difference (D)	−5.9
2022	United Republic of Tanzania	Difference (D)	−9.3
2022	Zambia	Difference (D)	−17.6
2022	Zimbabwe	Difference (D)	−15.2

Note: D is calculated as female prevalence minus male prevalence in the Health Equity Assessment Toolkit. Negative values indicated a higher male prevalence.

## Discussion

This multi-country descriptive analysis found wide variation in the 2022 modelled cigarette smoking prevalence and consistently higher estimates among males than females. The largest absolute sex gaps occurred in Lesotho, Mauritius, Algeria, Madagascar, and Seychelles. The finding that male prevalence exceeded female prevalence in all the included settings is consistent with the broader literature on tobacco use in Africa and globally [6,9,13,17].

The observed differences should not be interpreted as direct evidence of why smoking patterns vary. Prior studies have identified socioeconomic position, education, urban residence, and other population characteristics as correlates of tobacco use [8–11]. The literature has also proposed gender-related norms, stigma surrounding women’s smoking, and tobacco marketing as possible contributors to male–female differences [13–16]. These explanations are plausible hypotheses for future country-specific research, not mechanisms tested in the present analysis.

For policy, the results support routine reporting of male and female estimates rather than relying solely on national averages. Countries with a high male prevalence and large absolute gaps may require stronger implementation of population-wide tobacco control measures alongside interventions designed to reach men. Countries with a comparatively higher female prevalence should ensure that prevention and cessation services are accessible to women and that surveillance can detect changes over time. These recommendations should be adapted to local evidence rather than being inferred solely from cross-country rankings.

The analysis also illustrates the differences between absolute and relative inequality measures. Absolute gaps identify where the percentage-point burden differs most, whereas male-to-female ratios can become very large when the female prevalence is close to zero. Therefore, for this dataset, relative ratios should be interpreted alongside the absolute differences and the underlying sex-specific estimates rather than being used as a stand-alone ranking. The cross-country comparison is strengthened by the use of a standardized World Health Organization indicator and a common 2022 reference year. However, comparability does not eliminate differences in the amount, timing, and quality of source survey information available to the World Health Organization model for each country. Better documentation and regular collection of sex-disaggregated tobacco data are important for interpreting country estimates and monitoring policy effects.

### Limitations

This study had several limitations. First, the results are World Health Organization modelled estimates for a common 2022 reference year rather than direct estimates from surveys conducted in every country in 2022; the underlying survey years, instruments, and data density may vary. Second, the indicator is non-age-standardized; thus, differences in population age structure may affect comparisons. Third, source surveys commonly rely on self-reports and may understate smoking where social stigma is strong. Fourth, 15 African countries were excluded because complete sex-disaggregated estimates were unavailable, which limited the continental coverage. Fifth, the analysis only addressed cigarettes and did not capture other smoked tobacco, smokeless tobacco, or electronic cigarettes. Sixth, the prevalence ratios are unstable when female estimates are very low or rounded to zero. Finally, the study did not measure cultural norms, religion, marketing exposure, socioeconomic conditions, or policy implementation; these factors cannot be presented as causal explanations for the country patterns.

### Future directions

Future research should link standardized prevalence estimates with country-specific information on survey timing, age distribution, socioeconomic position, tobacco control implementation, and marketing exposure. Repeated analyses using successive World Health Organization reference years would help distinguish persistent patterns from changes over time. Qualitative and mixed-methods studies could examine gender-related norms and stigma without assuming that these mechanisms are uniform across countries.

Surveillance systems should consistently report sex-disaggregated estimates and document source data supporting modelled trends. Evaluations of tobacco control policies should use longitudinal or quasi-experimental designs where feasible because the cross-sectional comparisons in this study cannot estimate policy effects.

## Conclusion

This study provides a multi-country descriptive summary using standardized World Health Organization Global Health Observatory estimates accessed through the Health Equity Assessment Toolkit version 6.0. For the 2022 reference year, the prevalence of current cigarette smoking varied substantially across 39 African countries, and the male prevalence exceeded the female prevalence in every included country.

The findings support stronger sex-disaggregated surveillance and country-specific tobacco control planning, particularly in settings with high male prevalence or large absolute gaps. They did not establish that cultural norms, marketing, or policy differences caused the observed patterns. Future research should directly test these possible explanations and assess changes using repeated and policy-relevant data.

## Supplementary Material

Supplementary table 2.csv

Supplementary table 1.csv

## Availability data and materials

The exported datasets used for the analysis are provided with this submission. The underlying World Health Organization Health Equity Assessment Toolkit is publicly accessible at https://www.who.int/data/inequality-monitor/assessment_toolkit.

## References

[1] Siddiqi K, Husain S, Vidyasagaran A, et al. Global burden of disease due to smokeless tobacco consumption in adults: an updated analysis of data from 127 countries. BMC Med. 2020 18:1–8. doi: 10.1186/s12916-020-01677-9 32782007 PMC7422596

[2] Sinha DN, Suliankatchi RA, Gupta PC, et al. Global burden of all-cause and cause-specific mortality due to smokeless tobacco use: a systematic review and meta-analysis. Tob Control. 2018 27:35–42. 27903956 10.1136/tobaccocontrol-2016-053302

[3] Gupta S, Kumar V, Gupta P. A comprehensive study on the harmful effects of the smoking on human beings. In: Challenges in information, communication and computing technology [Internet]. 1st ed. London: CRC Press; 2024 [cited 2026 Sep 6]. p. 577–582. https://www.taylorfrancis.com/books/9781003559085/chapters/10.1201/9781003559085-99

[4] World Health Organization. WHO global report on trends in prevalence of tobacco use 2000–2030. World Health Organization. 2024.

[5] Jafari A, Rajabi A, Gholian-Aval M, et al. National, regional, and global prevalence of cigarette smoking among women/females in the general population: a systematic review and meta-analysis. Environ Health Prev Med. 2021 26:1–3. doi: 10.1186/s12199-020-00924-y 33419408 PMC7796590

[6] World Health Organization. WHO global report on trends in prevalence of tobacco smoking 2000–2025.

[7] Vellios N, Ross H, Perucic AM. Trends in cigarette demand and supply in Africa. PLOS ONE. 2018 13:e0202467. doi: 10.1371/journal.pone.0202467 30118508 PMC6097686

[8] Mahdaviazad H, Foroutan R, Masoompour SM. Prevalence of tobacco smoking and its socioeconomic determinants: tobacco smoking and its determinants. Clin Respir J. 2022 16:208–215. doi: 10.1111/crj.13470 35060332 PMC9060085

[9] Sreeramareddy CT, Pradhan PM, Sin S. Prevalence, distribution, and social determinants of tobacco use in 30 sub-Saharan African countries. BMC Med. 2014 12:1–3. doi: 10.1186/s12916-014-0243-x PMC4296681 25518855

[10] Sreeramareddy CT, Pradhan PM, Mir IA, et al. Smoking and smokeless tobacco use in nine South and Southeast Asian countries: prevalence estimates and social determinants from demographic and Health surveys. Popul Health Metrics. 2014 12:1–6. doi: 10.1186/s12963-014-0022-0 PMC4151025 25183954

[11] Casetta B, Videla AJ, Bardach A, et al. Association between cigarette smoking prevalence and income level: a systematic review and meta-analysis. Nicotine Tob Res. 2017 19:1401–1407. 27679607 10.1093/ntr/ntw266

[12] Taylor RS. Using statistical modelling approaches to explore the prevalence of cigarette smoking in the Global South [Doctoral dissertation]. University of Southampton; 2024.

[13] Islami F, Stoklosa M, Drope J, et al. Global and regional patterns of tobacco smoking and tobacco control policies. Eur Urol Focus. 2015 1:3–16. doi: 10.1016/j.euf.2014.10.001 28723352

[14] Amos A, Greaves L, Nichter M, et al. Women and tobacco: a call for including gender in tobacco control research, policy and practice. Tob Control. 2012 21:236–243. doi: 10.1136/tobaccocontrol-2011-050280 22166266

[15] Cruz TB, Rose SW, Lienemann BA, et al. Pro-tobacco marketing and anti-tobacco campaigns aimed at vulnerable populations: a review of the literature. Tob Induc Dis. 2019 17:68. doi: 10.18332/tid/111397 31582956 PMC6770621

[16] Hoek J, Edwards R, Waa A. From social accessory to societal disapproval: smoking, social norms and tobacco endgames. Tob Control. 2022 31:358–364. doi: 10.1136/tobaccocontrol-2021-056574 35241613

[17] Aboagye RG, Mohammed A, Duodu PA, et al. Sex-related inequalities in current cigarette smoking among adolescents in Africa. Substance abuse treatment. Subst Abuse Treat Prev Policy. 2024 19:41. doi: 10.1186/s13011-024-00619-5 39237953 PMC11375970

